# A panel of eGFP reporters for single base editing by APOBEC-Cas9 editosome complexes

**DOI:** 10.1038/s41598-018-36739-9

**Published:** 2019-01-24

**Authors:** A. St. Martin, D. J. Salamango, A. A. Serebrenik, N. M. Shaban, W. L. Brown, R. S. Harris

**Affiliations:** 1 Department of Biochemistry, Molecular Biology and Biophysics, University of Minnesota, Minneapolis, Minnesota 55455 USA; 2 Masonic Cancer Center, University of Minnesota, Minneapolis, Minnesota 55455 USA; 3 Institute for Molecular Virology, University of Minnesota, Minneapolis, Minnesota 55455 USA; 4 Center for Genome Engineering, University of Minnesota, Minneapolis, Minnesota 55455 USA; 5 Howard Hughes Medical Institute, University of Minnesota, Minneapolis, Minnesota 55455 USA

## Abstract

The prospect of introducing a single C-to-T change at a specific genomic location has become feasible with APOBEC-Cas9 editing technologies. We present a panel of eGFP reporters for quantification and optimization of single base editing by APOBEC-Cas9 editosomes. Reporter utility is demonstrated by comparing activities of seven human APOBEC3 enzymes and rat APOBEC1 (BE3). APOBEC3A and RNA binding-defective variants of APOBEC3B and APOBEC3H display the highest single base editing efficiencies. APOBEC3B catalytic domain complexes also elicit the lowest frequencies of adjacent off-target events. However, unbiased deep-sequencing of edited reporters shows that all editosomes have some degree of local off-target editing. Thus, further optimization is required to generate true single base editors and the eGFP reporters described here have the potential to facilitate this process.

## Introduction

The single-stranded DNA cytosine to uracil (C-to-U) deamination activity of several members of the antiviral APOBEC family has been harnessed recently for site-specific genome engineering by incorporation into Cas9/guide (g)RNA editing complexes^1–8^. An advantage of this technology over canonical Cas9 editing is precise single base substitution mutations (C-to-T) without potentially detrimental intermediates and outcomes including DNA double-stranded breaks (DSBs) and insertion/deletion mutations (indels). Efforts to improve this technology are ongoing and include the utilization of different wild-type and mutant APOBEC enzymes to improve specificity, Cas9 nickase to promote fixation of uracil lesions as mutations and prevent DSB formation, and uracil DNA glycosylase inhibitor (UGI) to prevent local uracil base excision and repair^1–4,9–13^. Despite these and other modifications, the current generations of editosomes still frequently mutate off-target cytosines and cause indels, which are both adverse events likely to impede translational goals of correcting genetic diseases (reviewed by refs^14–16^).

All base editing studies to date require DNA sequencing to quantify ratios of intended/on-target and unintended/off-target events. As a complement to this technical necessity, we developed a mCherry restoration-of-function assay that requires APOBEC-mediated DNA editing at two adjacent sites followed by DNA breakage and DSB repair by non-homologous end-joining^2^. Despite enabling quantification of real-time APOBEC editing activity in living cells, this assay necessarily requires multiple activities including DSBs that are undesirable for *bona fide* single base editing. Here, we report the development of a panel of reporter constructs in which a single on-target C-to-T editing event restores eGFP fluorescence and enables real-time quantification of on-target DNA editing.

## Results

**Figure 1:**
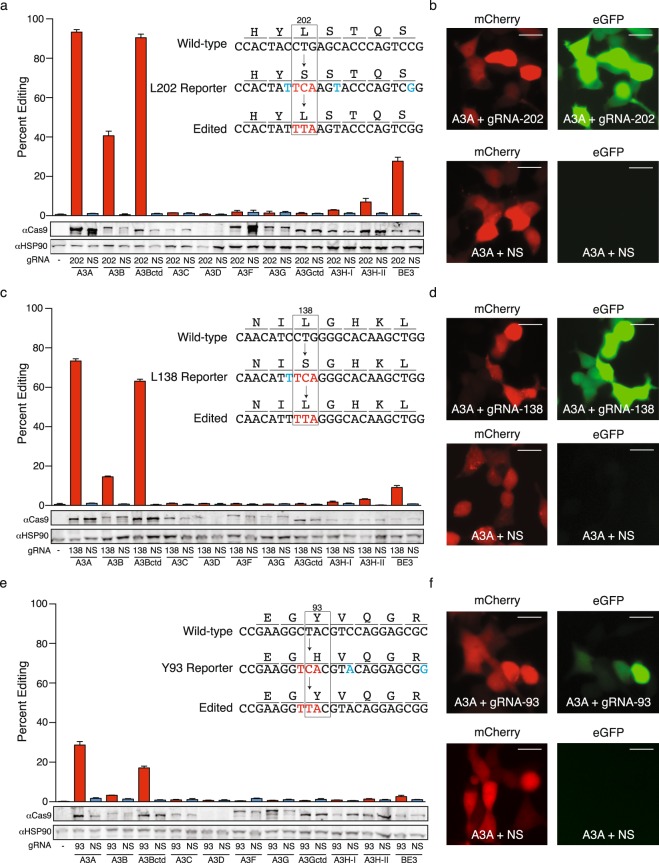
Editing efficiencies for episomal single base reporters. (**a**) Quantification of APOBEC editosome activities using the eGFP L202 single base editing reporter in 293 T cells (n = 3, average ± SD). Immunoblots are shown below for a representative experiment. Inset shows the wild-type eGFP codon 202 region, the mutated L202 reporter sequence, and the editing event required to restore eGFP activity. (**b**) Representative fluorescent microscopy images of 293 T cells transfected with the L202 reporter, the APOBEC3A editosome plasmid, and a gRNA-202 or a non-specific (NS) gRNA construct (scale bar = 20 µm). (**c**–**f**) Quantification of APOBEC editosome activities using eGFP L138 and Y93 single base editing reporters, respectively. Experiments as in panels a,b.

Three eGFP codons were identified where a T-to-C mutation ablates fluorescence and simultaneously creates a potential APOBEC editing site (L202, L138, and Y93 depicted in insets of Fig. 1a,c,e, respectively; Methods). One or more silent mutations were also purposely introduced alongside these specific changes in order to reduce the number of nearby editing sites, decrease the likelihood of DSBs, and optimize the PAM required for gRNA recognition. Each inactivated eGFP editing reporter is positioned downstream of a wild-type mCherry gene and a T2A site, which ensures efficient translation. The constitutively expressed upstream mCherry gene functions as a marker for assessing transfection and transduction efficiencies. Single base editing efficiencies are therefore quantified by dividing the fraction of eGFP and mCherry double-positive cells by the fraction of total mCherry-positive cells.

We first tested reporter utility by comparing efficiencies of single base editing in transiently transfected 293 T cells by the established rat APOBEC1 editosome (BE3)^1^, recently reported APOBEC3A and APOBEC3B C-terminal catalytic domain(ctd)-Cas9n-UGI complexes^17^, and new editosome constructs for APOBEC3B (full-length), APOBEC3C, APOBEC3D, APOBEC3F, APOBEC3G, and two naturally occurring variants of APOBEC3H (haplotype I and II) (Fig. 1). This panel spans the entire seven enzyme human APOBEC3 repertoire. For each editosome complex, efficiencies were highest for the L202 reporter, lower for the L138 reporter, and lowest for the Y93 reporter (Fig. 1a–f, respectively). Moreover, within a given reporter data set, APOBEC3A and APOBEC3Bctd editosomes showed the highest activity, followed by APOBEC3B (full-length), rat APOBEC1, and APOBEC3H-II. All other editosomes showed negligible activity, which may be based in part on poor expression (APOBEC3D), different dinucleotide editing preference (5′-CC, APOBEC3G), and/or as-yet-unknown reasons. DNA sequencing was not used to analyze these episomal DNA editing events due to a vast excess of non-edited reporter plasmid in each transient transfection reaction.

**Figure 2:**
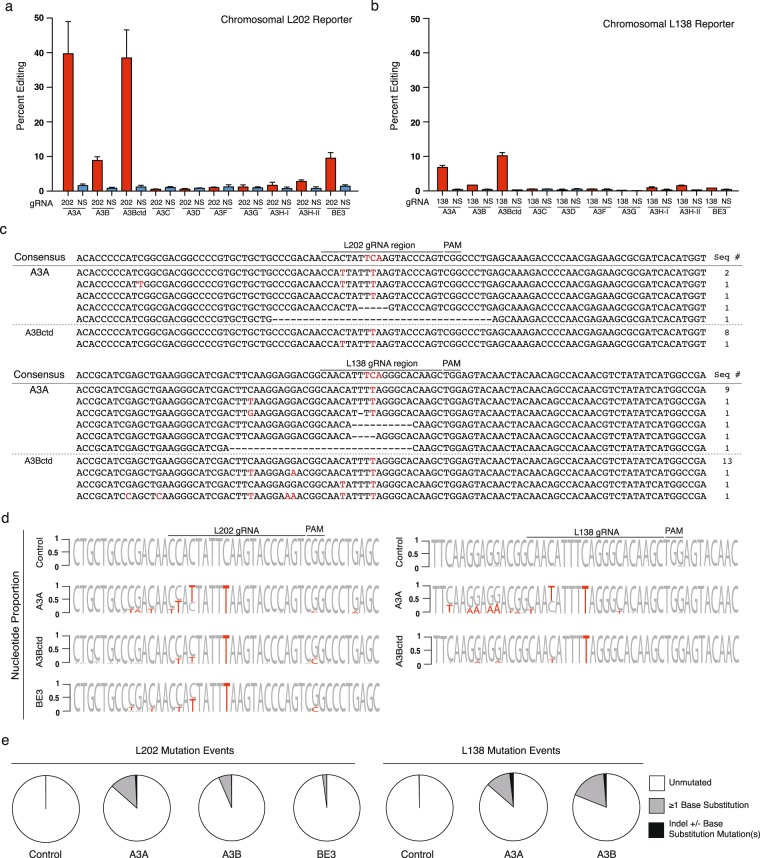
Editing efficiencies for chromosomal single base reporters. (**a**,**b**) Quantification of APOBEC editosome activities for chromosomally integrated eGFP L202 and L138 single base editing reporters in 293 T cells (n = 3, average ± SD). (**c**) Sanger sequences of the gRNA binding regions of the L202 and L138 reporters edited by APOBEC3A and APOBEC3Bctd editosomes in panel a. Single base substitutions are colored red. Deletions are represented by hyphens. The number of times each sequence was recovered is shown on the right. (**d**) Sequence logos summarizing deep sequencing data for the gRNA binding regions of the L202 and L138 reporters with on-target editing events. Base substitution mutations occurring in at least 5% of the reads are shown in red. The L202 PAM mutation is likely a PCR or MiSeq artifact due to G/C richness as it is also present in the control reactions. (**e**) Pie graphs showing MiSeq read proportions with no mutations (white), one or more single base substitutions (gray), or indels (black; some of which are also coincident with single base substitutions).

Next, chromosomal DNA editing efficiencies were compared by transiently co-transfecting each editosome construct and an appropriate eGFP gRNA into 293 T cell pools pre-engineered to contain a *single copy* of each editing reporter by lentivirus-mediated transduction (Fig. 2, Methods). For each editosome, the overall frequencies of eGFP-positive cells were lower than those for transiently transfected reporters, likely due in part to fewer editing substrates per cell (*i.e*., one versus many). However, relative editing and reporter efficiencies were still similar with APOBEC3A and APOBECBctd editing more efficiently than full-length APOBEC3B, BE3, and APOBEC3H-II, and the L202 reporter performing better than the L138 and Y93 reporters (Fig. 2a,b). In fact, Y93 chromosomal data were not shown because eGFP fluorescence rarely rose above background.

Sanger DNA sequencing was then used to assess mutational events in FACS-enriched, eGFP-positive cells. Due to enrichment by FACS (conservatively 85%), we anticipated finding a majority of on-target editing events and a minority of adjacent off-target edits and indels (*i.e*., additional mutational events within the DNA region analyzed by PCR and sequencing). However, only APOBEC3Bctd showed consistently high frequencies of on-target editing (8/9 for L202 and 13/16 for L138; Fig. 2c). In comparison, APOBEC3A showed lower than expected on-target editing events, with only 1/6 for the L202 reporter and 9/14 for the L183 reporter (Fig. 2c). Significant numbers of indels were also recovered in APOBEC3A reactions potentially due to imperfect FACS and/or preferential amplification of shorter DNA fragments by PCR.

These results were confirmed and extended by deep-sequencing the portion of each eGFP reporter that spans the intended editing target site (Methods). First, we noted that the overall frequency of on-target editing events reflects the proportion of eGFP-positive, reporter-activated cells in the overall mCherry-positive cell population (data not shown). Second, we used these unbiased deep-sequencing data sets to ask what frequencies and types of adjacent off-target base substitution mutations are observed alongside the on-target C-to-T editing events (Fig. 2d). Not surprisingly, the highly active APOBEC3A enzyme catalyzed the highest proportion of adjacent off-target events in both the L202 and L138 reporters with, for instance, >50% C-to-T at the position 5 nucleotides upstream of the intended target and high frequencies at other editing sites further upstream. APOBEC3A also caused mutations outside of the gRNA-targeted region (*i.e*., upstream of the single-stranded DNA in the R-loop created by gRNA annealing) indicating that this upstream DNA can become single-stranded at some frequency through different mechanisms such as transcription or DNA replication. BE3 editosomes also caused significant off-target events both within and upstream of the R-loop, whereas APOBEC3Bctd editosomes caused fewer overall off-target events and most of these were confined to the 5′-end of the R-loop. In all instances, relatively few off-target mutations were observed downstream of the intended target cytosine. Similar observations have been made previously using BE3 at several different target sites (*e.g*., refs^1,18–20^).

**Figure 3:**
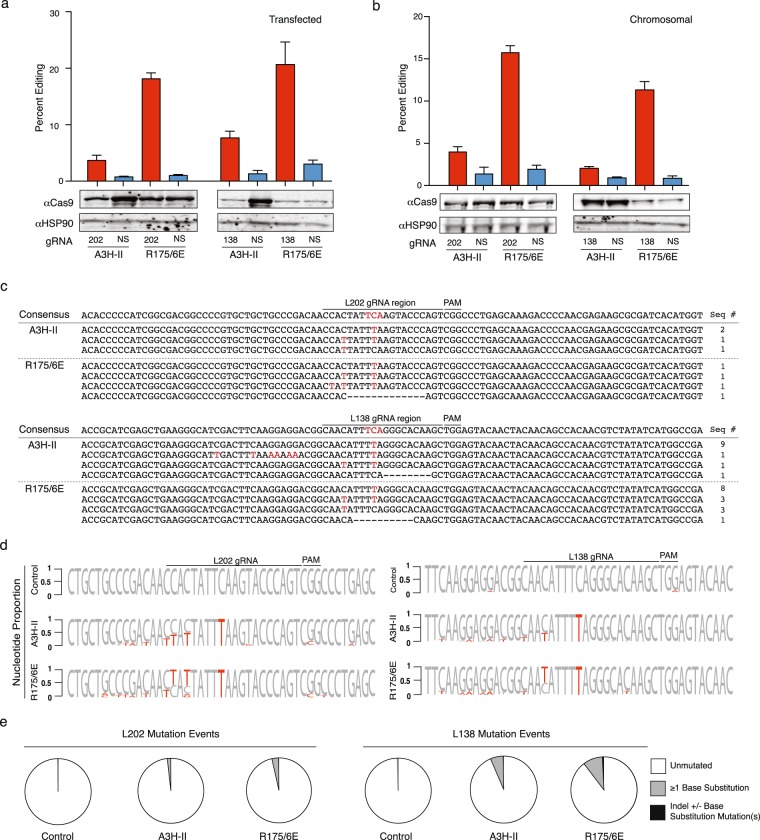
Single base editing in DNA is inhibited by RNA binding. (**a**,**b**) L202 and L138 reporter quantification of the single base editing activity of wild-type APOBEC3H (haplotype II) versus a RNA binding-defective mutant (R175/6E) (n = 3, average ± SD). Immunoblots below for a representative experiment. (**c**) Sanger sequences of the gRNA binding regions of the L202 and L138 reporters edited by APOBEC3H editosomes in panel a. Single base substitutions are colored red. Deletions are represented by hyphens. The number of times each sequence was recovered is shown on the right. (**d**) Sequence logos summarizing deep sequencing data for the gRNA binding regions of the L202 and L138 reporters with on-target editing events. Base substitution mutations occurring in at least 5% of the reads are shown in red. The L202 PAM mutation is likely a PCR or MiSeq artifact due to G/C richness as it is also present in the control reactions. (**e**) Pie graphs showing MiSeq read proportions with no mutations (white), one or more single base substitutions (gray), or indels (black; some of which are also coincident with single base substitutions).

Full-length APOBEC3B has two canonical deaminase domains, a catalytically active C-terminal domain and an inactive N-terminal domain known to bind RNA^21–23^. The higher base editing activity of APOBEC3Bctd in comparison to full-length APOBEC3B suggested that RNA binding might somehow interfere with single base editing (*e.g*., a bound bulky RNA may prevent the catalytic site from accessing target cytosines in single-stranded DNA). To test this idea directly, we used human APOBEC3H-II, which was recently shown to bind RNA through a basic patch distinct from its DNA editing active site^24,25^. Substitution of two adjacent arginines to glutamates (R175E/R176E) disrupts the RNA binding activity of APOBEC3H-II and increases its single-stranded DNA editing activity^24^. A comparison of the single base editing activity of APOBEC3H-II editosomes and an otherwise identical R175E/R176E RNA binding mutant showed that the mutant is 3.1- to 5.5-fold more active regardless of whether the reporter is episomal or chromosomal (Fig. 3a,b). Sanger and MiSeq DNA sequencing showed similar levels of on-target editing events for each APOBEC3H editosome complex, but adjacent off-target events occurred at higher frequencies for the hyperactive RNA binding-defective enzyme (Fig. 3c,d). Both constructs also caused indels but at lower frequencies than APOBEC3A (Fig. 3e).

## Discussion

This study describes the first fluorescent reporters for real-time quantification of single base editing by APOBEC-Cas9 editosomes in living cells. These eGFP reporters enabled us to perform the first comprehensive analysis of base editing capabilities of the entire seven protein human APOBEC3 repertoire. A detailed understanding of why some APOBEC enzymes are highly efficient DNA editors (APOBEC3A and APOBEC3Bctd), some are intermediate (rat APOBEC1, full-length APOBEC3B and APOBEC3H-II), and others are poor will be important for developing optimized editors for specific fundamental, applied, and biomedical applications. For instance, the RNA binding activity of APOBEC3H is clearly inhibitory and, therefore, strategies to eliminate or lessen this activity without compromising DNA editing activity may be beneficial. Many other variables may also influence single base editing efficiencies including Cas9 on/off rates, Cas9 endonuclease activity, linker length/composition, construct size, overall editosome solubility, subcellular localization, and as-yet-unidentified cellular factors that interact with APOBEC3 enzymes in human cells (*e.g*., refs^26–29^).

Reporter and editosome constructs described here could also be used, among many conceivable applications, to identify active variants of otherwise dead editosomes (reporter-up screen of editosome mutant libraries), variants of existing editosomes with increased single base selectivity (reporter-up screen with Y93 construct that currently yields modest eGFP fluorescence due to stop codon creation by adjacent off-target editing of codon 95), and cellular regulators of single base editing (CRISPR screens for reporter-up and -down mutants identifying negative and positive regulators, respectively). The local context of the target cytosine (5′-TCA in eGFP reporters described here) could also be altered to 5′-CCA, 5′-ACA, or 5′-GCA (or moved to different codon positions as necessary) to screen for mutant editosomes with different di- and tri-nucleotide preferences (*e.g*., 5′-TC to 5′-CC in ref.^30^). The eGFP reporters described here may also be easily adapted for use in a wide variety of different cellular systems (animal, plant, bacterial, parasite, *etc*.).

## Methods

### Single base editing reporters

The dual fluorescent HIV-based parental vector was reported^2^ (pLenti-CMV-mCherry-T2A-eGFP). Single base editing reporters were made by replacing wild-type eGFP with mutant eGFP PCR products made by overlapping extension high-fidelity PCR with Phusion DNA polymerase (NEB) using primers listed in Supplementary Table 1. Full-length PCR products were gel purified, digested with *Xho*I and *Kpn*I, and ligated into a similarly cut parental vector. The resulting L202, L138, and Y93 single base editing reporters were confirmed by diagnostic restriction digestions and Sanger sequencing.

### APOBEC editosome constructs

The rat APOBEC1-Cas9n-UGI-NLS construct (BE3) was provided by David Liu^1^. APOBEC cDNA sequences were amplified using primers in Supplementary Table 1 and high-fidelity PCR using previously validated Harris lab collection plasmids as templates. GenBank accession numbers for APOBEC3A, APOBEC3B, APOBEC3C, APOBEC3D, APOBEC3F, APOBEC3G, and APOBEC3H-II are, respectively, KM266646.1, AY743217.1, NM_014508, NM_152426, NM_145298, NM_021822, and NM_181773^21,24,31,32^. The resulting PCR products were cut with *Not*I and *Xma*I and used to replace rat APOBEC1 in BE3 (*Not*I site in MCS and *Xma*I site in XTEN linker). The gRNAs targeting L202, L138, and Y93 in eGFP or non-specific (NS) sequence as a control were synthesized as complementary oligonucleotides (Supplementary Table 1) and cloned into MLM3636, obtained from J. Keith Joung through Addgene (plasmid #43860), using the accompanying Joung Lab gRNA cloning protocol.

### Episomal base editing experiments

Semi-confluent 293 T cells in a 6-well plate format were transfected with 200 ng gRNA, 400 ng reporter, and 600 ng of each base editor [10 min at RT with 6 µl of TransIT LT1 (Mirus) and 200 µl of serum-free DMEM (Hyclone)]. Cells were harvested following 72 hrs incubation for editing quantification by flow cytometry.

### Chromosomal base editing experiments

Semi-confluent 10 cm plates of 293 T cells were transfected with 8 μg of an HIV-1 Gag-Pol packaging plasmid, 1.5 μg of a VSV-G expression plasmid, and 3 μg of each base editing reporter. Viruses were harvested 48 hrs post-transfection and used to transduce target cells (MOI = 0.1). 48 hrs post-transduction cells were sorted to enrich for a mCherry-positive population (confirmed >85% by subsequent flow cytometry and fluorescence microscopy). Transduced, mCherry-positive cells were transfected with 800 ng APOBEC-Cas9n-UGI editor and 200 ng of targeting or NS-gRNA were transfected into a semi-confluent 6-well plate of reporter-transduced cells. Cells were harvested 72 hrs post-transfection and editing was quantified by flow cytometry (fraction of eGFP and mCherry double-positive cells in total mCherry-positive population).

In a subset of chromosomal editing experiments, eGFP-positive cells were recovered by FACS, converted to genomic DNA (Qiagen Gentra Puregene), and subjected to high-fidelity PCR using Phusion (NEB) to amplify eGFP target sequences. PCR products were gel-purified (GeneJET Gel Extraction Kit, Thermo Scientific) and cloned into a sequencing plasmid (CloneJET PCR Cloning Kit, Thermo Fisher). Sanger sequencing was done in 96-well format (Genewiz) using primers recommended with the CloneJET PCR Cloning Kit (Supplementary Table 1).

To perform MiSeq experiments, eGFP target sequences were amplified using primers in Supplementary Table 1 and Phusion high-fidelity DNA polymerase (NEB). To add diversity to the sequence library, zero, one, or two extra cytosine bases were added to forward and reverse primers for each amplicon. Barcodes were added to generate full-length Illumina amplicons. Samples were analyzed using Illumina MiSeq (University of Minnesota Genomics Center) 2 × 75-nucleotide paired-end reads. Reads were paired using FLASh^33^. Data processing was performed using a locally installed FASTX-Toolkit. Fastx-clipper was used to trim the 3′ constant adapter region from sequences, and a stand-alone script was used to trim 5′ constant regions. Trimmed sequences were then filtered for high-quality reads using the Fastx-quality filter. Sequences with a Phred quality score less than 30 (99.9% base calling accuracy) at any position were eliminated. Preprocessed sequences were then further analyzed using the FASTAptamer toolkit^34^. FASTAptamer-Count was used to determine the number of times each sequence was sampled from the population. Each sequence was then ranked and sorted based on overall abundance, normalized to the total number of reads in each population, and directed into FASTAptamer-Enrich. FASTAptamer-Enrich calculates the fold enrichment ratios from a starting population to a selected population by using the normalized reads-per-million (RPM) values for each sequence. Sequences at abundances lower than 5 RPM in the A3-editosome samples were discarded. For reporter and A3-editosome comparisons, sequences that appeared only in the A3-contianing samples (with an RPM value over 5), or, sequences that occurred at a frequency below 5 RPM in the No-editor control were included for analysis.

### Immunoblots

1 × 10^6^ cells were lysed directly into 2.5x Laemmli sample buffer, separated by 8% SDS-PAGE, and transferred to PVDF-FL membranes (Millipore). Membranes were blocked in 5% milk in PBS and incubated with primary antibody diluted in 5% milk in PBS supplemented with 0.1% Tween20. Secondary antibodies were diluted in 5% milk in PBS supplemented with 0.1% Tween20 and 0.01% SDS. Membranes were imaged with a Licor Odyssey instrument. Primary antibodies used in these experiments were rabbit anti-Cas9 (Abcam ab204448) and mouse anti-HSP90 (BD Transduction Laboratories 610418). Secondary antibodies used were goat anti-rabbit IRdye 800CW (Licor 827-08365) and goat anti-mouse Alexa Fluor 680 (Molecular Probes A-21057). Relevant regions of each immunoblot are shown in Figs 1 and 3, and full images are provided in the supplement.

## Electronic supplementary material

Supplementary Figure 1 and Full Immunoblots

## Data Availability

The datasets generated during and/or analyzed during the current study are available from the corresponding author on reasonable request.
