# Supplementary material for: A panel of eGFP reporters for single base editing by APOBEC-Cas9 editosome complexes
Source: Sci Rep. 2019 Jan 24;9:497. doi: 10.1038/s41598-018-36739-9 (PMC6345908; doi:10.1038/s41598-018-36739-9)

Supplementary Information for

A panel of eGFP reporters for single base editing by APOBEC-Cas9 editosome complexes

A. St. Martin[1,2,3,4], D.J. Salamango[1,2,3,4], A.A. Serebrenik[1,2,3,4], N.M. Shaban[1,2,3,4], W.L. Brown[1,2,3,4], R.S. Harris[1,2,3,4,5,*]

[1] Department of Biochemistry, Molecular Biology and Biophysics, University of Minnesota, Minneapolis, Minnesota, USA, 55455.

[2] Masonic Cancer Center, University of Minnesota, Minneapolis, Minnesota, USA, 55455.

[3] Institute for Molecular Virology, University of Minnesota, Minneapolis, Minnesota, USA, 55455.

[4] Center for Genome Engineering, University of Minnesota, Minneapolis, Minnesota, USA, 55455.

[5] Howard Hughes Medical Institute, University of Minnesota, Minneapolis, Minnesota, USA, 55455.

[*] Correspondence should be addressed to RSH (rsh@umn.edu).

Contents:

Supplementary Figure 1

Images of full immunoblots corresponding to Figure 1a/c/e and Figure 3a/b

Supplementary Table 1. Oligonucleotides used in this study.

Description	Sequence (5' to 3')
Y93H Mutation Forward	CCATGCCCGAAGGTCACGTACAGGAGCGGACCATCTTC
Y93H Mutation Reverse	GAAGATGGTCCGCTCCTGTACGTGACCTTCGGGCATGG
L138S Mutation Forward	GGACGGCAACATTTCAGGGCACAAGCTGGA
L138S Mutation Reverse	TCCAGCTTGTGCCCTGAAATGTTGCCGTCC
L202S Mutation Forward	CGACAACCACTATTCAAGTACCCAGTCGGCCCTGA
L202S Mutation Reverse	TCAGGGCCGACTGGGTACTTGAATAGTGGTTGTCG
Full-length A3Bi cloning primer forward	AGATCC-GCGGCCGC-GCCGCCACCATG-AATCCACAGATCAGAAATCCGATGG
Full-length A3Bi cloning primer reverse	TGAGGTCCCGGGAGTCTCGCTGCCGCTGTTTCCCTGATTCTGGAGAATGGCC
A3C cloning primer forward	**AGATCC**GCGGCCGC**GCCGCCACCATG**AATCCACAGATCAGAAACCCGATGA
A3C cloning primer reverse	TGAGGTCCCGGGAGTCTCGCTGCCGCTCTGGAGACTCTCCCGTAGCCTTCTT
A3D cloning primer forward	AGATCCGCGGCCGCGCCGCCACCATGAATCCACAGATCAGAAATCCGATGG
A3D cloning primer reverse	TGAGGTCCCGGGAGTCTCGCTGCCGCTCTGGAGAATCTCCCGTAGCCTTCTT
A3F cloning primer forward	AGATCCGCGGCCGCGCCGCCACCATGAAGCCTCACTTCAGAAACACAGTGG
A3F cloning primer reverse	TGAGGTCCCGGGAGTCTCGCTGCCGCTCTCGAGAATCTCCTGCAGCTTGCTG
A3G cloning primer forward	AGATCCGCGGCCGCGCCGCCACCATGAAGCCTCACTTCAGAAACACAGTGG
A3G cloning primer reverse	TGAGGTCCCGGGAGTCTCGCTGCCGCTGTTTTCCTGATTCTGGAGAATGGCC
A3H-I and A3H-II cloning primer forward	AGATCCGCGGCCGCGCCGCCACCATGGCTCTGTTAACAGCCGAAACATTCCG
A3H-i and A3H-II cloning primer reverse	TGAGGTCCCGGGAGTCTCGCTGCCGCTTCAGGACTGCTTTATCCTGTCAAGC
GFP Y93H gRNA forward	**ACACC**CCGAAGGTCACGTACAGGA**G**
GFP Y93H gRNA reverse	**AAAAC**CTCCTGTACGTGACCTTCGGG
GFP L138S gRNA forward	**ACACC**CAACATTTCAGGGCACAAGC**G**
GFP L138S gRNA reverse	**AAAAC**GCTTGTGCCCTGAAATGTTGG
GFP L202S gRNA forward	**ACACC**CCACTATTCAAGTACCCAGT**G**
GFP L202S gRNA reverse	**AAAAC**ACTGGGTACTTGAATAGTGG**G**
CloneJET Sequencing Forward Primer	CGACTCACTATAGGGAGAGCGGC
CloneJET Sequencing Reverse Primer	AAGAACATCGATTTTCCATGGCAG
L138 Miseq Forward No C	acactctttccctacacgacgctcttccgatctTCGAGCTGAAGGGCATCGAC
L138 Miseq Forward One C	acactctttccctacacgacgctcttccgatctCTCGAGCTGAAGGGCATCGAC
L138 Miseq Forward Two C	acactctttccctacacgacgctcttccgatctCCTCGAGCTGAAGGGCATCGAC
L138 Miseq Reverse No C	gtgactggagttcagacgtgtgctcttccgatctTAGACGTTGTGGCTGTTG

	TA
L138 Miseq Reverse One C	gtgactggagttcagacgtgtgctcttccgatctGTAGACGTTGTGGCTGTT GTA
L138 Miseq Reverse Two C	gtgactggagttcagacgtgtgctcttccgatctGGTAGACGTTGTGGCTGT TGTA
L202 Miseq Forward No C	acactctttccctacacgacgctcttccgatctCCATCGGCGACGGCCCCGT G
L202 Miseq Forward One C	acactctttccctacacgacgctcttccgatctCCCATCGGCGACGGCCCCG TG
L202 Miseq Forward Two C	acactctttccctacacgacgctcttccgatctCCCCATCGGCGACGGCCCC GTG
L202 Miseq Reverse No C	gtgactggagttcagacgtgtgctcttccgatctCGCTTCTCGTTGGGGTCT TT
L202 Miseq Reverse One C	gtgactggagttcagacgtgtgctcttccgatctGCGCTTCTCGTTGGGGTC TTT
L202 Miseq Reverse Two C	gtgactggagttcagacgtgtgctcttccgatctGGCGCTTCTCGTTGGGGT CTTT

Full immunoblots for Figure 1a

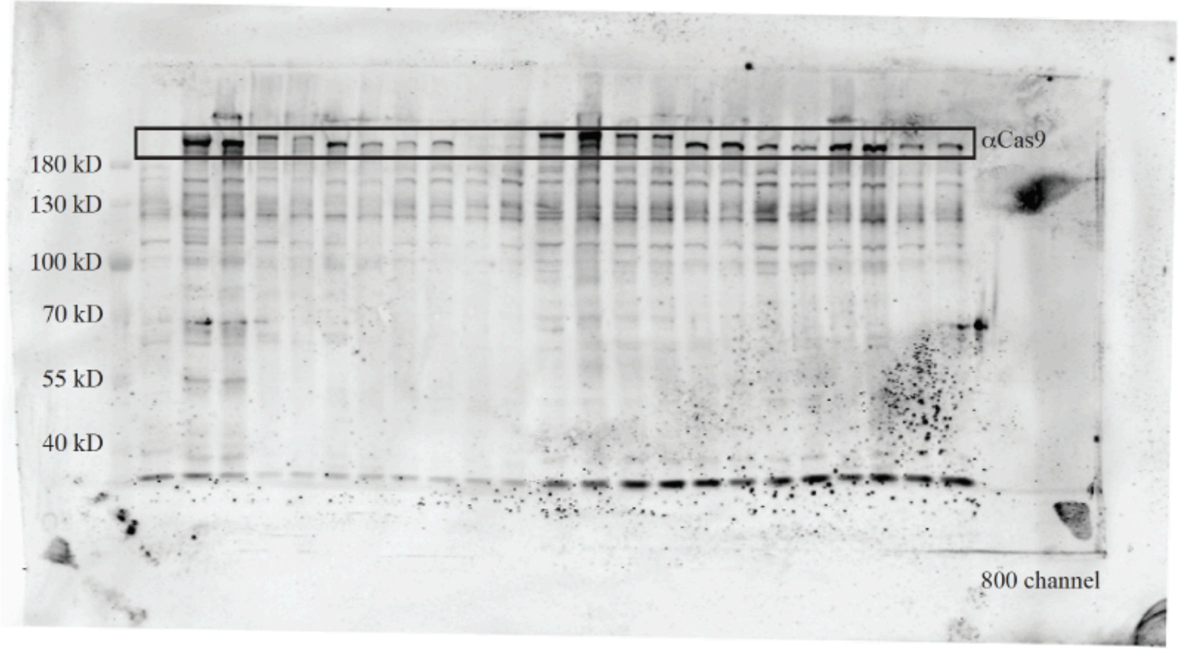

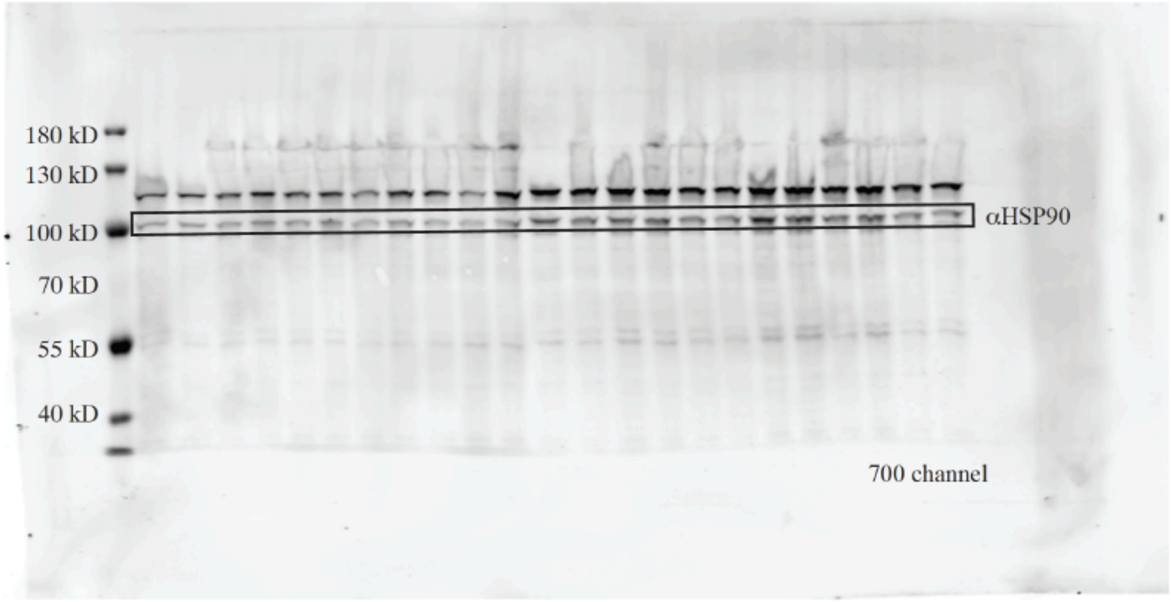

Full immunoblots for Figure 1b

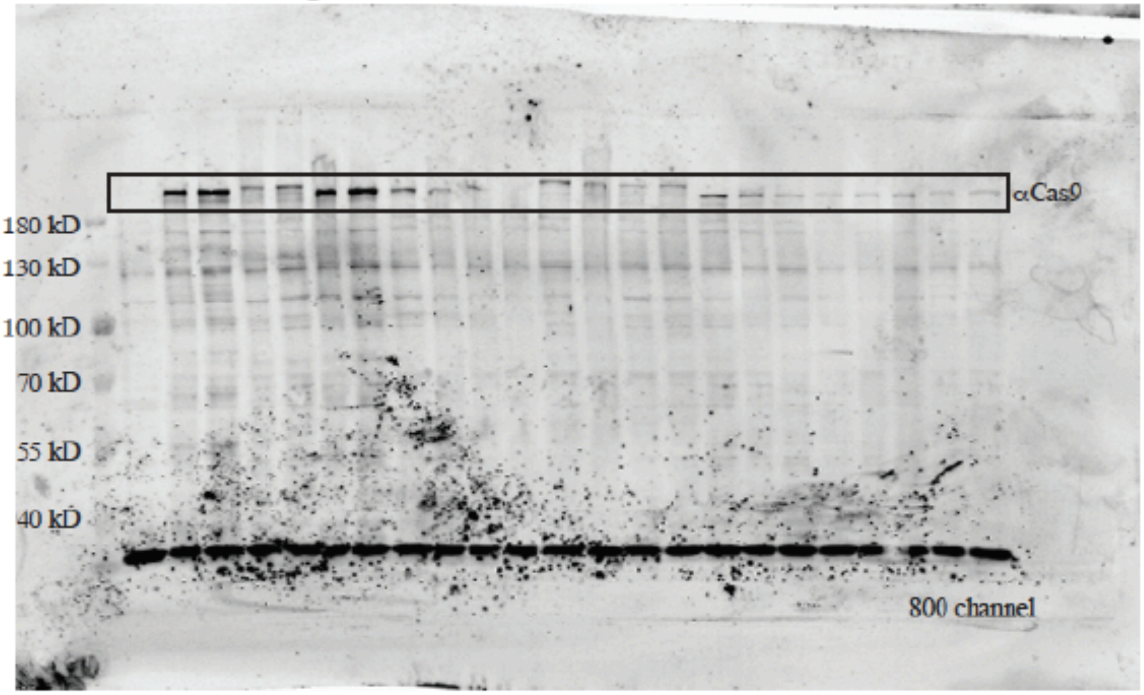

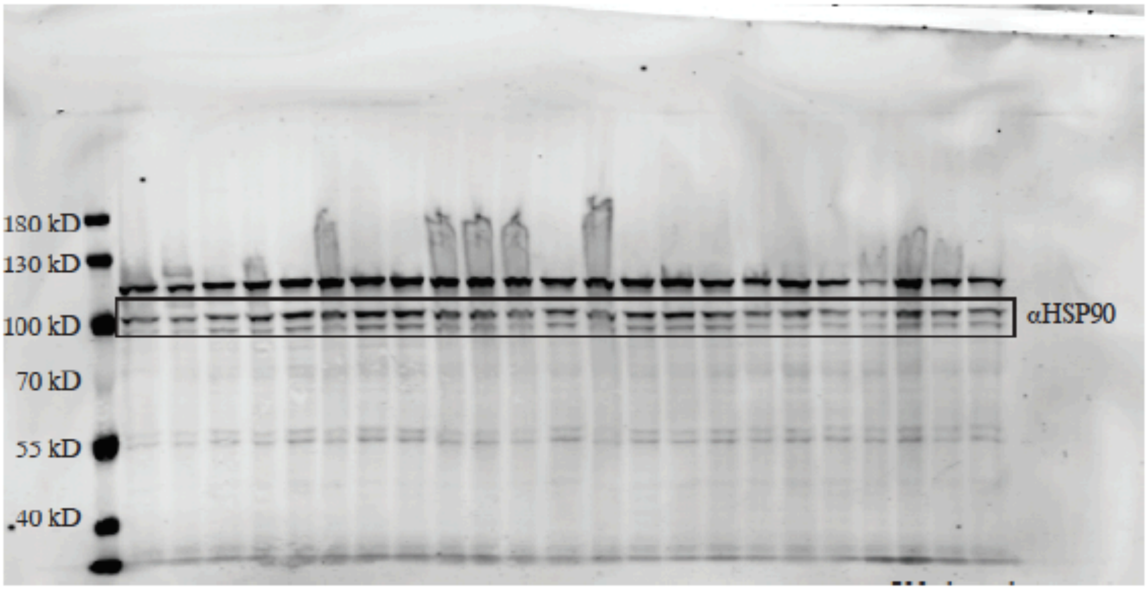

700 Channel

Full immunoblots from Figure 1c

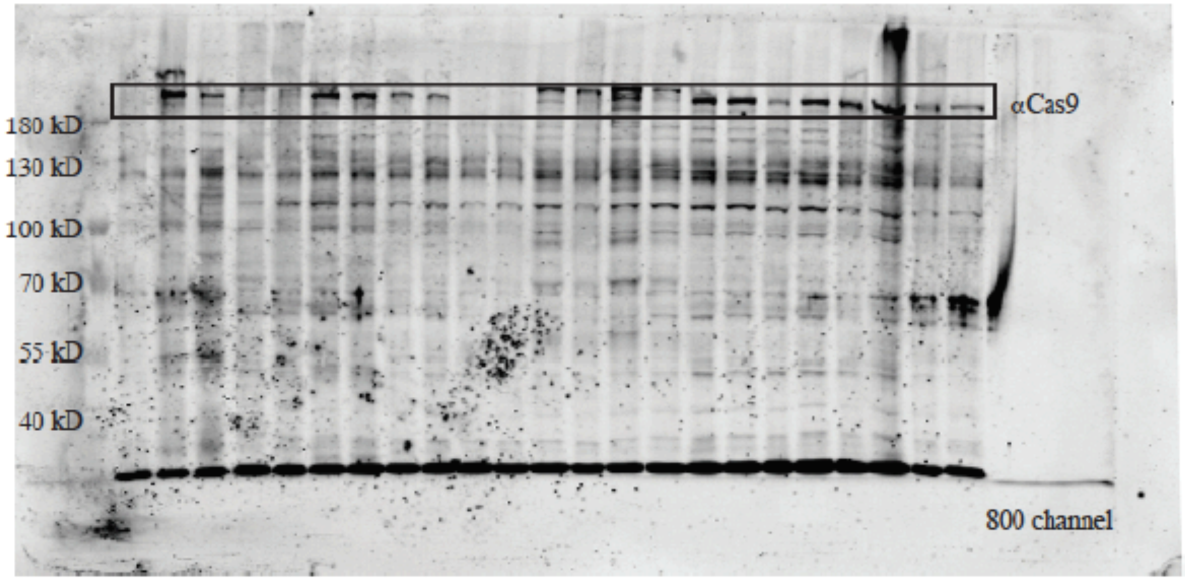

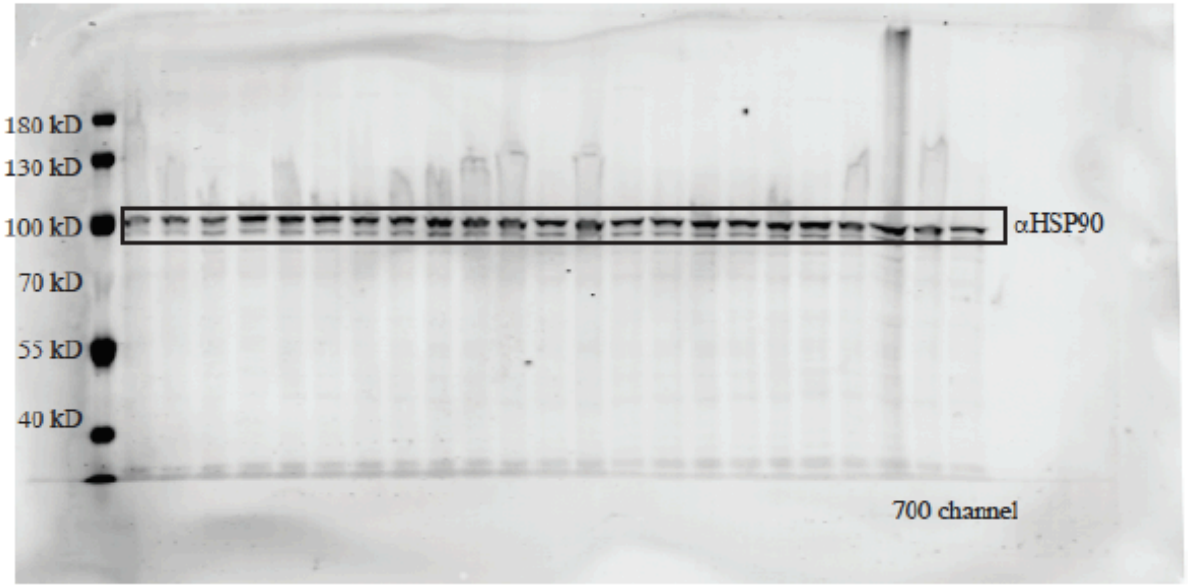

Full blot images Figure 3a and 3b

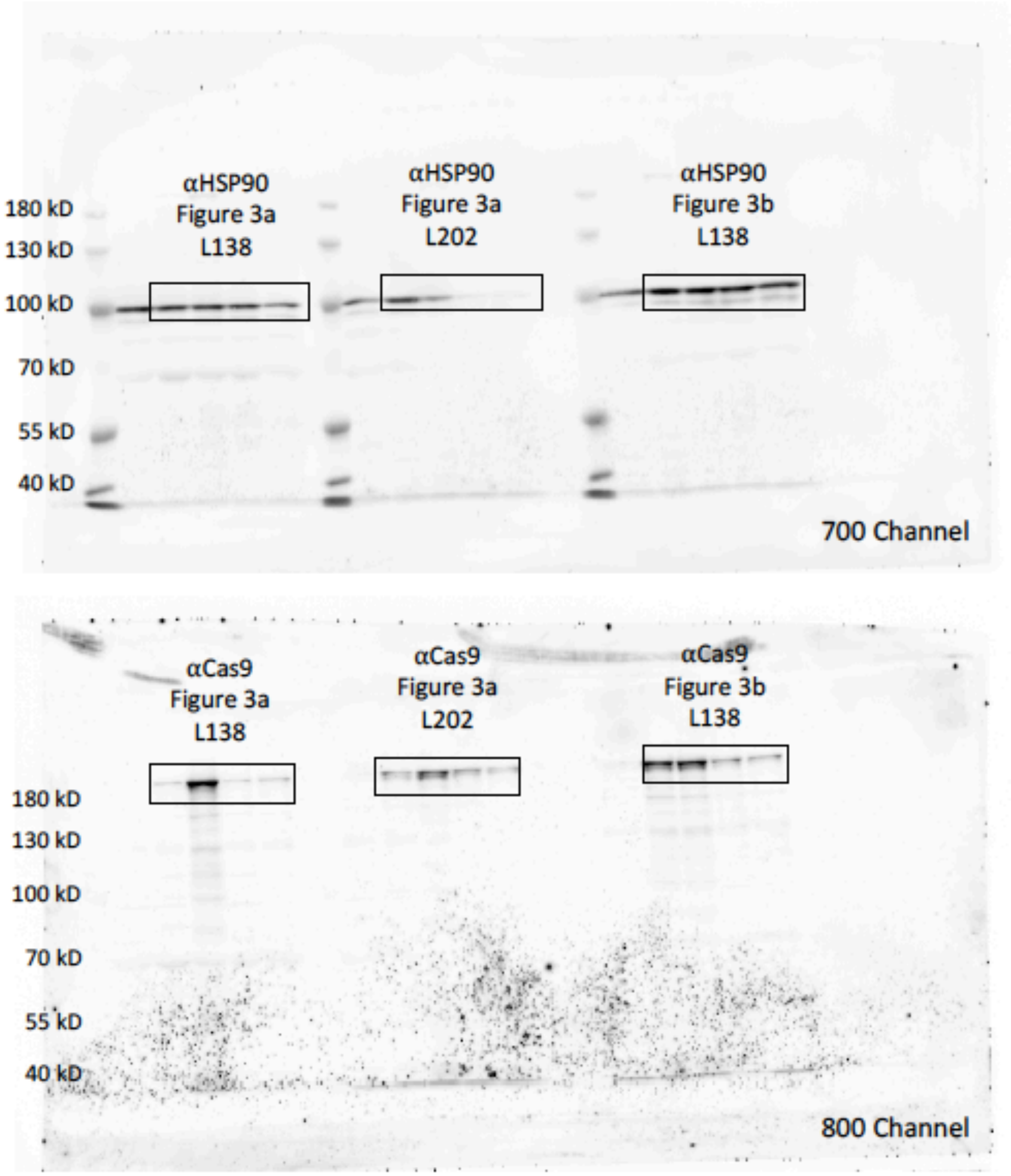

Full immunoblots Figure 3b L202

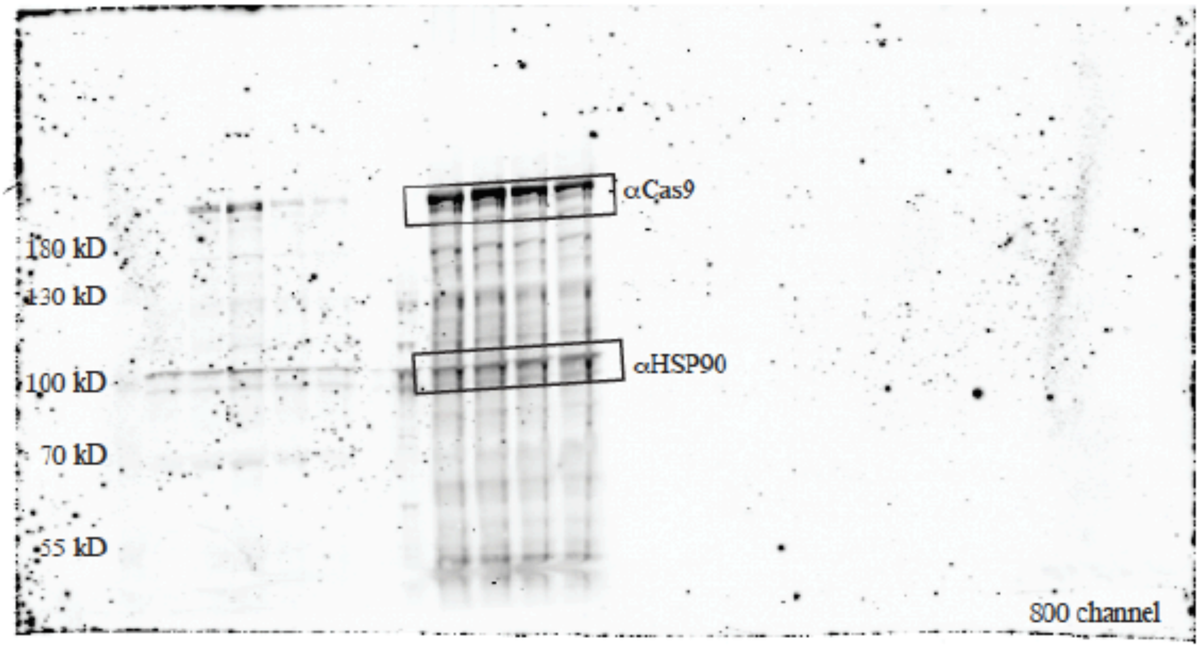

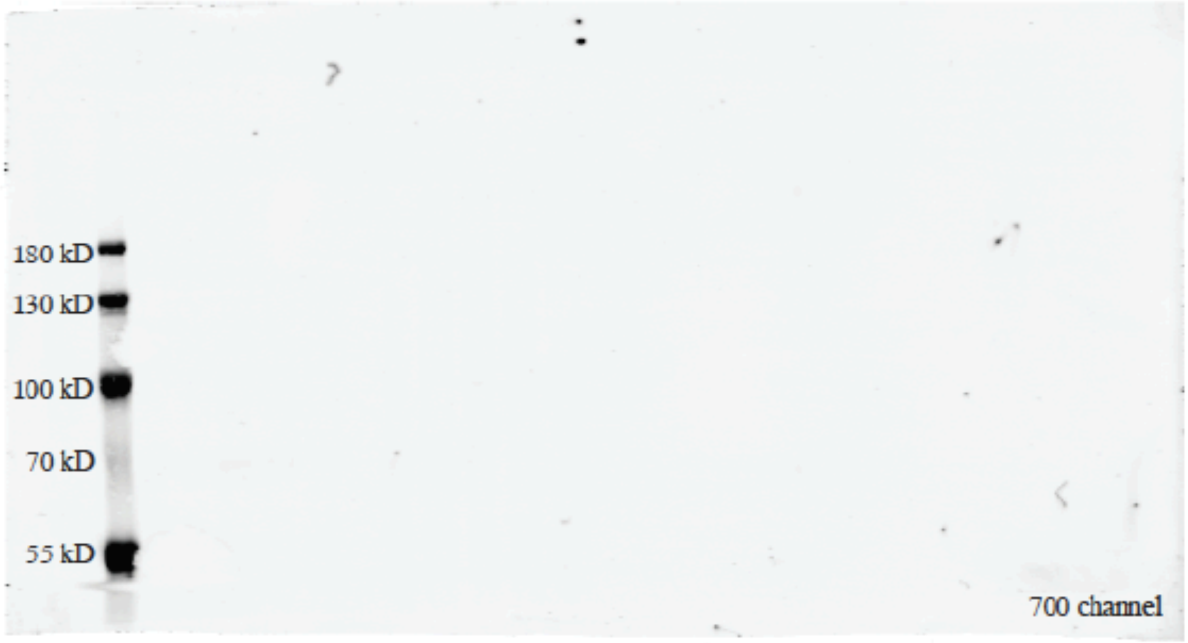

Supplement: Supplementary file 1 — Supplementary Figure 1 and Full Immunoblots [file 41598_2018_36739_MOESM1_ESM.pdf]
